# A new instrument for estimation of survival in elderly patients irradiated for metastatic spinal cord compression from breast cancer

**DOI:** 10.1186/s13014-015-0483-8

**Published:** 2015-08-19

**Authors:** Dirk Rades, Antonio J. Conde, Raquel Garcia, Jon Cacicedo, Barbara Segedin, Ana Perpar, Steven E. Schild

**Affiliations:** Department of Radiation Oncology, University of Lübeck, Ratzeburger Allee 160, 23538 Lübeck, Germany; Department of Radiation Oncology, Consorcio Hospital Provincial de Castellón, Castellón, Spain; Department of Radiation Oncology, Cruces University Hospital, Barakaldo, Vizcaya Spain; Department of Radiotherapy, Institute of Oncology Ljubljana, Ljubljana, Slovenia; Department of Radiation Oncology, Mayo Clinic, Scottsdale, AZ USA

## Abstract

**Background:**

Elderly patients become more important in oncology. In this group, personalized treatment approaches taking into account survival prognoses and comorbidities play a major role. Predictive instruments are necessary to estimate the survival of elderly cancer patients. The importance of separate instruments for different tumor entities has been recognized. In this study, an instrument was generated to estimate the survival of elderly patients developing metastatic spinal cord compression (MSCC) from breast cancer.

**Methods:**

In 218 elderly patients (age ≥65 years) irradiated for MSCC from breast cancer, nine factors were evaluated for survival: fractionation regimen, age, time from breast cancer diagnosis to RT of MSCC, visceral metastases, other bone metastases, time developing motor deficits, pre-radiotherapy ambulatory status, number of involved vertebrae, and Eastern Cooperative Oncology Group (ECOG) performance score. Factors significantly associated with survival in the Cox regression analysis were included in the prognostic instrument. Scores for each factor were calculated by dividing the 6-months survival rates by 10. The sums of these scores represented the patients’ scores.

**Results:**

On multivariate analyses, visceral metastases (*p* < 0.001), time developing motor deficits (*p* < 0.001), ambulatory status (*p* < 0.001), number of involved vertebrae (*p* = 0.032), and ECOG performance score (*p* < 0.001) were significant and included in the prognostic instrument. Based on the patients’ scores, three groups were designed: 18–27 points, 28–39 points and 40–42 points. Six-months survival rates were 4, 62 and 100 %, respectively (*p* < 0.001).

**Conclusions:**

This new instrument contributes to personalized treatment in elderly patients with MSCC from breast cancer by predicting an individual patient’s survival prognosis.

## Background

Due to demographic changes, the proportion of elderly patients is constantly increasing in many countries. This is also true for patients requiring oncologic treatment. Therefore, these cancer patients need particular attention in terms of tailored treatment regimens taking into account an individual patient’s performance status, comorbidities and survival prognosis [1–4]. The latter can be estimated with applying predictive instruments. Due to improved medical support and treatment of non-oncologic diseases in many countries worldwide, more elderly cancer patients live longer. Since the risk of developing metastases increases with lifetime, the numbers of patients with metastases are growing. A considerable number of these patients present with metastatic spinal cord compression (MSCC), which can occur in up to 10 % of all adult cancer patients [5, 6]. Therefore, elderly patients with MSCC need more attention, and personalized treatment is very important. In order to optimize such personalized approaches, the patients’ survival prognoses must be considered. Therefore, it is mandatory to have predictive instruments available that are specifically designed for elderly patients. A survival score for elderly patients with MSCC has already been created [7]. However, that score has been developed in a cohort of patients with MSCC from many different primary tumor types. It is already recognized that primary tumor types differ with respect to tumor biology, metastatic patterns and prognoses. Therefore, separate scores have been created for several tumor entities [8–12]. However, these scores did not consider the special characteristics of elderly cancer patients. Therefore, instruments predicting survival are required for elderly patients that focus on a single primary tumor type. In the current study, such an instrument has been developed particularly for elderly patients with MSCC from breast cancer. This new instrument will contribute to further optimization of personalized treatment of elderly breast cancer patients.

## Patients and methods

Two-hundred-and-eighteen elderly (age: 65 years or older) patients with MSCC from breast cancer were included in this retrospective study, which was approved by the local ethic committee. The fractionation regimen (1 × 8 Gy vs. 5 × 4 Gy vs. 10 × 3 Gy vs. 15 × 2.5 Gy vs. 20 × 2 Gy) plus eight additional factors were evaluated with respect to potential associations with survival. These additional factors were age (≤73 vs. ≥74 years, median age 73 years), time from breast cancer diagnosis to RT of MSCC (≤15 vs. >15 months), presence of visceral metastases prior to RT (no vs. yes), presence of other bone metastases prior to RT (no vs. yes), time developing motor deficits prior to the start of RT (1–7 vs. 8–14 vs. <14 days), ambulatory status prior to RT (no vs. yes), number of involved vertebrae (1–3 vs. ≥4), and Eastern Cooperative Oncology Group (ECOG) performance score (1–2 vs. 3 vs. 4). Activities of daily living (ADL) were not added as a potential prognostic factor, since the ECOG performance score considers this aspect. Therefore, the addition of a score of ADL would have introduced confounding variables and redundant information. This applies also to the factor comorbidity. In the Charlson Comorbidity Index, metastatic cancer, which all of the patients included in our score were suffering from, was given the highest score [13]. Hemiplegia was also an important comorbidity in the Charlson Index, which is more or less reflected by ambulatory status (weakness of the legs, paraplegia) in our survival score.

Associations of the nine factors with survival were initially investigated in a univariate manner with the Kaplan-Meier method and the log rank test [14]. Those factors that achieved significance (*p* < 0.05) were subsequently analyzed for independence with the Cox regression analysis. Since pre-RT ambulatory status and performance status can be considered confounding variables, two Cox regression analyses were performed, one including the ambulatory status and one including the performance status. The factors, which remained significantly associated with survival also in the Cox regression, were included in the prognostic instrument. The score for each factor was calculated by dividing the corresponding 6-months survival rates by 10. The sum of these score scores represented the score for a patient.

## Results

**Fig. 1:**
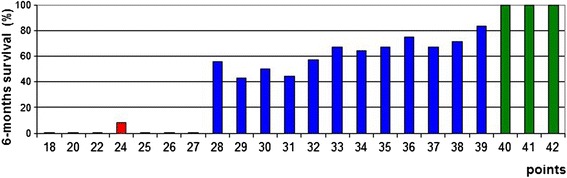
Sum scores for each patient and the corresponding 6-months survival rates

**Fig. 2:**
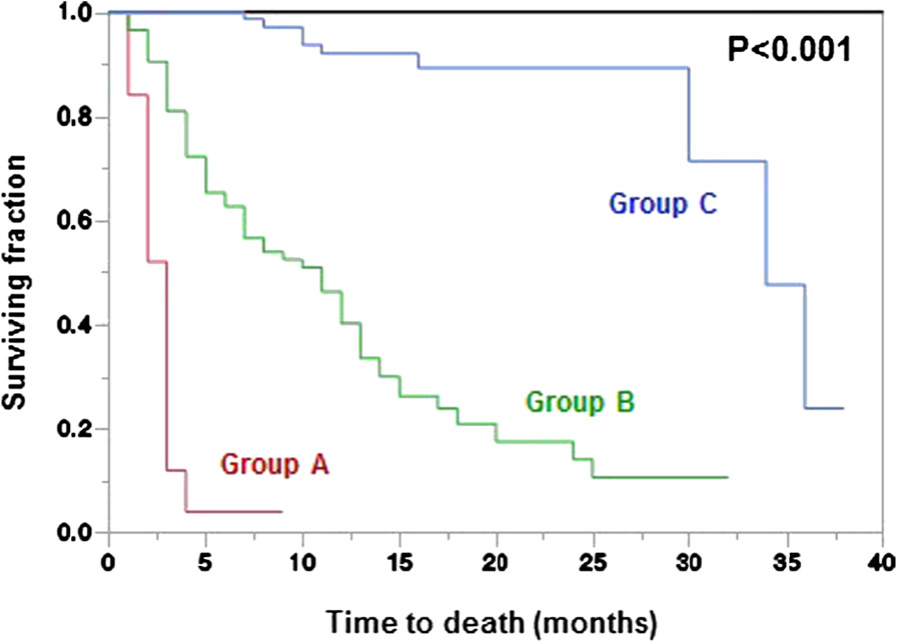
Kaplan-Meier curves for survival of the three prognostic groups A (18–27 points), B (28–39 points) and C (40–42 points)

In the univariate analysis, survival was positively influenced by absence of visceral metastases (*p* < 0.001), absence of other bone metastases (*p* < 0.001), slower development of motor deficits before the start of RT (*p* < 0.001), ambulatory status before RT (*p* < 0.001), involvement of only 1–3 vertebrae (*p* < 0.001), and an ECOG performance score of 1–2 (*p* < 0.001). The results of the univariate analyses are summarized in Table 1. In the subsequent Cox regression analyses, visceral metastases (*p* < 0.001), time developing motor deficits before the start of RT (*p* < 0.001), ambulatory status before RT (*p* < 0.001), number of involved vertebrae (*p* = 0.032), and the ECOG performance score (*p* < 0.001) maintained significance (Table 2). These five factors were included in the prognostic instrument for prediction of survival (Table 3). The sum scores for the patients ranged between 18 and 42 points. The 6-months survival rates related to these points are presented in Fig. 1. Taking into account these survival rates, three survival groups were designed, 18–27 points (group A), 28–39 points (group B) and 40–42 points (group C). The 6-months survival rates of these three groups were 4, 62 and 100 %, respectively (*p* < 0.001, Fig. 2). The p-values for the comparisons of group A vs. group B and group B vs. group C were *p* < 0.001 and *p* = 0.003, respectively.

**Table 1:** Impact of the investigated factors on survival (univariate analysis)

	At 6 months	At 12 months	P
Age			
≤ 73 years (*N* = 118)	69	57	
≥ 74 years (*N* = 100)	70	53	0.63
Interval from breast cancer diagnosis to RT of MSCC			
≤ 15 months (*N* = 56)	64	46	
> 15 months (*N* = 162)	71	58	0.23
Visceral metastases at the time of RT			
No (*N* = 117)	88	78	
Yes (*N* = 101)	48	28	<0.001
Other bone metastases at the time of RT			
No (*N* = 79)	82	65	
Yes (*N* = 139)	62	50	<0.001
Time developing motor deficits prior to RT			
1–7 days (*N* = 48)	33	6	
8–14 days (*N* = 70)	73	61	
> 14 days (*N* = 110)	83	70	<0.001
Ambulatory status prior to RT			
Not ambulatory (*N* = 66)	41	28	
Ambulatory (*N* = 152)	82	68	<0.001
Involved vertebrae (N)			
1–3 (*N* = 127)	79	66	
≥ 4 (*N* = 91)	56	40	<0.001
ECOG Performance status			
1–2 (*N* = 120)	87	73	
3 (*N* = 86)	55	38	
4 (*N* = 12)	0	0	<0.001
Fractionation regimen			
1 × 8 Gy (*N* = 26)	77	49	
5 × 4 Gy (*N* = 59)	66	58	
10 × 3 Gy (*N* = 72)	67	53	
15 × 2.5 Gy (*N* = 20)	65	65	
20 × 2 Gy (*N* = 41)	76	63	0.78

**Table 2:** Cox regression analysis of the factors that were significantly associated with survival in the univariate analysis

	Risk ratio	95 %-confidence interval	P
Visceral metastases at the time of RT			
no vs. yes	5.42	3.34 – 9.05	<0.001
Other bone metastases at the time of RT			
no vs. yes	1.35	0.80 – 2.27	0.26
Time developing motor deficits prior to RT			
> 14 days vs. 8–14 days vs. 1–7 days	1.68	1.30 – 2.17	<0.001
Ambulatory status prior to RT			
ambulatory vs. not ambulatory	2.34	1.56 – 3.51	<0001
Involved vertebrae (N)			
1–3 vs. ≥ 4	1.18	1.01 – 1.37	0.032
ECOG Performance status			
1–2 vs. 3 vs. 4	2.40	1.64 – 3.53	<0.001

**Table 3:** Six-months survival rates of the independent prognostic factors and the corresponding scoring points

	Survival at 6 months (%)	Scoring points
Visceral metastases at the time of RT		
No (*N* = 117)	88	9
Yes (*N* = 101)	48	5
Time developing motor deficits prior to RT		
1–7 days (*N* = 48)	33	3
8–14 days (*N* = 70)	73	7
> 14 days (*N* = 110)	83	8
Ambulatory status prior to RT		
Not ambulatory (*N* = 66)	41	4
Ambulatory (*N* = 152)	82	8
Involved vertebrae (N)		
1–3 (*N* = 127)	79	8
≥ 4 (*N* = 91)	56	6
ECOG Performance status		
1–2 (*N* = 120)	87	9
3 (*N* = 86)	55	6
4 (*N* = 12)	0	0

## Discussion

Elderly cancer patients are becoming more and more important, since there proportions and numbers are constantly increasing. In comparison to younger patients, elderly patients are different. They generally have a poorer performance status, a greater comorbidity index, a worse immune system, and a shorter survival. Therefore, when treating these patients, specific precautions are mandatory. In order to provide the best possible treatment for elderly cancer patients, very personalized approaches are needed. Such personalization should consider an individual patient’s remaining lifetime, which can be estimated with the help of predictive instruments. Because primary tumors are different, each tumor requires separate prognostic tools. In this study, we have created a scoring system to properly estimate the survival of elderly patients with MSCC from breast cancer. According to a recursive partitioning analysis for another metastatic situation, brain metastases, “elderly” patients were defined 65 years or older [15].

The present study identified five factors that were independent predictors of survival, visceral metastases, time developing motor deficits, ambulatory status, number of involved vertebrae (*p* = 0.032), and ECOG performance score. These five factors were included in the predictive instrument. When compared to the survival score developed in elderly patients with MSCC from different primary tumors, the independent factors of the present instrument were different [7]. In the previous scoring system, eight factors were independently associated with survival including age, performance status, tumor entity, pre-RT ambulatory status, other bone metastases, visceral metastases, interval from cancer diagnosis to radiotherapy of MSCC, and time developing motor deficits prior to RT. In comparison to the new score for elderly patients with MSCC from breast cancer, four additional factors were predictive for survival in that previous score. The number of involved vertebrae was significant in the new but not in the previous score. These differences demonstrate that each tumor entity must be considered separately and requires separate scoring systems. The independent prognostic factors in this study of elderly patients with MSCC from breast cancer also differed from the predictors of survival of another previous score created in patients with MSCC from breast cancer of any age [11]. Independent factors in that previous score included performance status, ambulatory status, other bone metastases, visceral metastases, interval from cancer diagnosis to radiotherapy of MSCC and time developing motor deficits, not the number of involved vertebrae. These differences support the need for separate scores for elderly cancer patients.

In the present study, three predictive groups with significantly different survival prognoses were identified: 18–27 points (group A), 28–39 points (group B) and 40–42 points (group C). In group A, only 4 % of the patients survived at least 6 months. To avoid that these patients spend unnecessarily too much of their very limited life time with treatment, they should be treated with single-fraction RT such as 1 × 8 Gy or multi-fraction short-course RT such as 5 × 4 Gy in one week. These fractionation regimens are not inferior to longer-course RT programs with respect to functional outcomes [16]. Patients of group B have an intermediate survival prognosis and can, therefore, be considered appropriate candidates for the worldwide most commonly used fractionation regimen, 10 × 3 Gy in two weeks, because 10 × 3 Gy results in better local control of MSCC than single-fraction RT with 1 × 8 Gy or short-course RT with 5 × 4 Gy in one week [17, 18]. Since patients of group C have a much more favorable survival prognosis with a 6.months survival probability of 100 %, these patients should receive longer-course RT with total doses greater than 30 Gy. In a previous study of patients with MSCC from different tumor types and a favorable survival prognosis, doses beyond 30 Gy resulted in significantly better local control of MSCC, progression-free survival and overall survival when compared to 10 × 3 Gy [19]. Selected patients of the prognostic groups B and C may be considered for decompressive surgery in addition to conventional radiotherapy or for stereotactic body radiation therapy instead of conventional RT, preferably within clinical trials [20–22].

In conclusion, this new predictive instrument contributes significantly to proper estimation of the survival of elderly patients with MSCC from breast cancer, and facilitates the administration of personalized treatment regimens in this particular group of cancer patients.
